# Frontline Mongolian Healthcare Professionals and Adverse Mental Health Conditions During the Peak of COVID-19 Pandemic

**DOI:** 10.3389/fpsyg.2022.800809

**Published:** 2022-03-11

**Authors:** Basbish Tsogbadrakh, Enkhjargal Yanjmaa, Oyungoo Badamdorj, Dorjderem Choijiljav, Enkhjargal Gendenjamts, Oyun-erdene Ayush, Odonjil Pojin, Battogtokh Davaakhuu, Tuya Sukhbat, Baigalmaa Dovdon, Oyunsuren Davaasuren, Azadeh Stark

**Affiliations:** ^1^School of Nursing, Mongolian National University of Medical Sciences, Ulaanbaatar, Mongolia; ^2^Inova Fairfax Hospital, Falls Church, VA, United States; ^3^General Hospital Darkhan-Uul, Darkhan, Mongolia; ^4^School of Medicine, Mongolian National University of Medical Sciences, Ulaanbaatar, Mongolia; ^5^Henry Ford Health System, Detroit, MI, United States; ^6^School of Interdisciplinary Studies, The University of Texas at Dallas, Richardson, TX, United States; ^7^Faculty of Nursing, Chiang Mai University, Chiang Mai, Thailand

**Keywords:** depression, anxiety, stress, psychological wellbeing, self-efficacy, insomnia, COVID-19

## Abstract

**Background:**

The relatively young and inexperienced healthcare professionals in Mongolia faced with an unprecedent service demand in response to the COVID-19 pandemic. Due to the small size of the healthcare workforce the Mongolian Health Ministry had no choice but to mandate continuous and long workhours from the healthcare workforce. Many of the healthcare professionals exhibited signs and symptoms of mental health disorders. This study aimed to discern the prevalence various mental health concerns, i.e., depression, anxiety and stress, insomnia, and to discern the factors that increased susceptibility to mental health disorders among frontline healthcare professionals providing healthcare services for COVID-19 patients in Mongolia.

**Methods:**

A Cross-sectional research design was implemented. We collected data from 965 healthcare professional, randomly selected from 18 government hospitals, in four regions of Mongolia. Data were collected using the Depression Anxiety Stress-21, the General Self-Efficacy Scale, and the Insomnia Severity Index instruments. We constructed the scale of Pandemic Response Symptoms (PaReSy) which captured stress, depression, and anxiety. Data were analyzed using descriptive statistics, Kruskal–Wallis statistical test and multinominal logistic regression analysis.

**Results:**

Prevalence of depression (52.3%, CI 95%: 49.1–55.5%), anxiety (70.2%, CI 95%: 67.2–73.0%), and stress (35.8%, CI 95%: 32.7–38.9%) was documented among Mongolian healthcare professionals. Perception of self-efficacy reduced susceptibility to PaReSy either at mild/moderate (OR = 0.948, 95% CI = 0.911–0.988, *P* = 0.011) or severe/extremely severe level (OR = 0.911, 95% CI = 0.861–0.963, *P* = 0.001). Within each stratum of insomnia, the risk of experiencing PaReSy increased almost linearly both in the category of mild/moderate PaReSy and in the category of severe/extremely severe PaReSy.

**Conclusion:**

Improving self-efficacy and sleeping quality can assist healthcare workers to manage depression, anxiety, and stress. Findings provide important evidence to implement measures and strategies to assist healthcare professionals in low- and middle-income countries to constructively address their mental health concerns and needs.

## Introduction

The pandemic of 2020–2021 has put an unprecedented burden on the healthcare workforce and healthcare infrastructure across the globe, but particularly in low- and middle-income countries (Hopman et al., 2020). This unprecedented burden, although multifactorial, partially can be attributed to the austerity measures that were implemented in response to the global economic crisis of 2008 (Gené-Badia et al., 2012; Reeves et al., 2014). Governments and private sectors imposed financial austerities and health policies and procedures to reduce the cost while improving the cost-effectiveness of delivery of healthcare services (Buchan et al., 2013). These changes put more emphasis and demand on the role and responsibilities of the healthcare force, which propagated the epidemic of burnout among the healthcare professionals and therefore exodus of many from the field (Mattei et al., 2017). Concerns about the shortage of healthcare professionals, across the spectrum of expertise, in 21st century were addressed extensively before the 2020 pandemic (Lipstein and Kellermann, 2016; World Health Organization, 2021a). The gravity of COVID-19 pandemic on the healthcare sector was exacerbated because of the shortage of healthcare workforce. World Health Organization (2021b) has highlighted the excessive burden of COVID-19 on healthcare force and has called for urgent action to address the basic needs and measures necessary to ameliorate the impact of pandemic on physical and mental wellbeing of healthcare workforce.

Despite the decisive actions of the government of Mongolia, the country did not remain immune to the pandemic. The State Emergency Committee and the Ministry of Health reported the first verified COVID-19 case of community transmission on November 11/2020 (Situation Report for Covid-19, 2020). The precipitous increase in the incidence of COVID-19 was experienced after March 2021 (Covid-19 Situation Report for Mongolia #58, 2021). The impact of COVID-19 on Mongolian society has been severe due to the limited number of healthcare professionals; for example, a total of 13,112 nursing professionals constitutes the population of the nursing workforce throughout the country (Mongolia Center for Health Development, 2019). Meanwhile, the current pandemic has had multiple mental and psychological side effects, i.e., depression, anxiety, or stress on the Mongolian healthcare professionals. Our professional concerns and ethical obligations required us to systematically and scientifically assess and document prevalence of various mental and psychological side effects of the COVID-19 pandemic among Mongolian healthcare professionals. This study aimed to discern the mental health of Mongolian frontline healthcare workers during the peak of COVID-19 pandemic.

## Materials and Methods

### Setting

Mongolia, a country of 3.3 million population, is bordered by China and Russia. The country, which is largely a plateau, with the average elevation of 5,180 feet above sea level, enjoys continental climate. About half of the population of Mongolia (1.6 million, or 48%) reside in Ulaanbaatar, the capital of Mongolia. The city which sits in the Tuul River valley (surrounded by Khangai and Khentii mountain regions) is vulnerable to the meteorological phenomenon of thermal inversion, particularly during the cold season. Ulaanbaatar is susceptible to various respiratory diseases, due to its high population density, air pollution that is caused by fumes of car exhaust systems and coal burning heating devices and the repeated thermal inversions (Chimeddorj et al., 2021). The first case of COVID-19 in Mongolia was documented in November 11/2020 in the city of Ulaanbaatar; by March 2021, the entire country was impacted, despite the four mandated lock downs and facial mask requirements (Covid-19 Situation Report for Mongolia #62, 2021).

In Mongolia the healthcare policy of eight beds per 1,000 population were stipulated, based on data from healthcare services utilization and econometric analysis, to address the hospital-base healthcare needs of the Mongolian population. However, during the peak of COVID-19 pandemic, in the capital city the bed occupancy rate in the intensive care units reached to 96.1% and the overall hospital bed occupancy was at 98.9% (Covid-19 Situation Report for Mongolia #58, 2021). In response, the Emergency Commissions of the State and the Capital City assigned the Ulaanbaatar Health Department to open additional hospital beds for emergency use. The Health Department along with district hospitals responded by supplying additional 1,200 beds and the necessary medical equipment, i.e., ventilating machines, oxygen supplies, to address the pandemic. In collaboration with UNICEF Mongolia, Ministry of Health (MoH) and Mongolian Red Cross Society (MRCS) distributed disinfectants, hygiene supplies and PPEs to 1,410 frontline workers, positioned at border controls and high-risk areas (Covid-19 Situation update, Mongolia Update#12, 2021). On 28 October, 2020, additional PPEs were procured through the Luxembourg Cooperation Grant and were delivered to the community hospitals throughout Mongolia (Situation Report #4, 2020 on COVID-19 response). Despite the efforts of the Mongolian government and the support of the international community, the demand on for healthcare services far exceeded the supplies and the personnel; the frontline workers, were experiencing longer working hours, more than usual workload and, without any doubt, they were concerned about their own and their family members health and wellbeing; all of these created the optimal milieu for mental duress and psychological stress.

### Study Design

This was a descriptive cross-sectional study to investigate the prevalence various mental health concerns, i.e., depression, anxiety and stress, insomnia, and to discern the factors that increased susceptibility to mental health disorders among frontline healthcare professionals providing healthcare services at different hospitals designated for COVID-19 patients in Mongolia.

### Population and Sample

**FIGURE 1:**
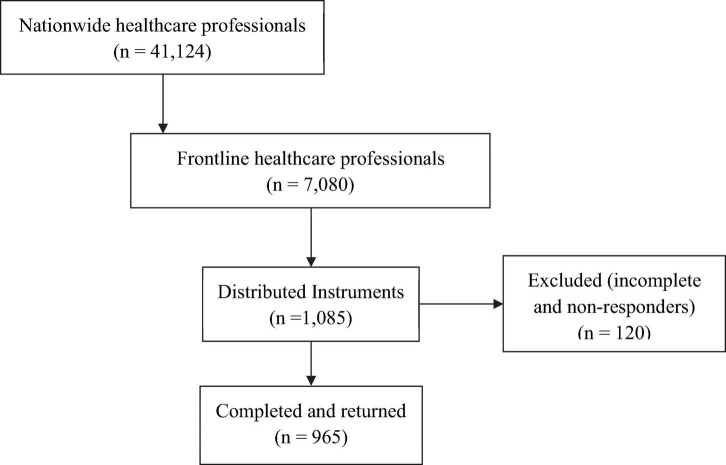
Flow diagram of recruitment of study participants and distribution of the study survey instruments.

The total number of 7,080 healthcare professionals working on the frontline to combat COVID-19 pandemic in Mongolia was used to estimate the sample size for our study (Mongolia Center for Health Development, 2021). We applied the Hsieh sample size formula for logistic regression (Hsieh, 1989) to calculate the sample size of our study; our calculation yielded a sample size of 1,085 (inflated 20% for non-responders) healthcare professionals [α = 0.05, odds ratio (OR) = 1.3, power of the test (1-β) = 0.80] who were recruited from 18 hospitals, owned, and operated by the government of Mongolia. We applied proportional stratified random sampling technique to adjust for the size of healthcare professionals in each hospital (Burns and Grove, 2010; Table 1 and Figure 1).

**TABLE 1:** Number of study participants stratified by participating hospital.

Province	Name of hospital	Population of healthcare professionals	Sample size
Ulaanbaatar city	Military Hospital	298	36
	General Hospital for Special Civil Servants	323	39
	First State Central Hospital	824	99
	Second State Central Hospital	564	68
	Third State Central Hospital	800	96
	National Center for Communicable Diseases	750	90
	National Center for Maternal and Child Health of Mongolia	800	96
	First Maternity Hospital	282	78
	Mongolian Japanese Teaching Hospital, MNUMS	362	53
	National Dermatology Center of Mongolia	79	11
	Songinokhairkhan District Health Center	324	60
	Hospital for Injury and Trauma	812	98
	Bayangol District Health Center	300	79
	Chingeltei District Health Center	300	36
	Khan Uul District Hospital	324	39
Regional hospitals	Khovd, Khuvsgul Aimag Regional Diagnostic and Treatment Center (Western Region Hospital)	722	124
	Darkhan, Orkhon Aimag Regional Diagnostic and Treatment Center (Central Region Hospital)	837	109
	Dornod Aimag Regional Diagnostic and Treatment Center (Eastern Region Hospital)	300	60

### Eligibility Criteria

Healthcare professionals were eligible to participate in our study if: (1) They were frontline healthcare workers during the peak of COVID-19 pandemic; (2) They were actively employed by the government hospital system; (3) They were willing to participate in our study. We excluded healthcare professionals who had participated in the pre-testing stage of our study; we imposed this restriction to reduce the likelihood of information bias.

### Research Instrument

The first section of our instrument was designed to capture demographic data, i.e., age, gender, level of education, occupation, and years of experience in the healthcare sector. The General Self-Efficacy Scale (GSES) constituted the second section (Schwarzer and Jerusalem, 1995). In this section, our focus was to measure the confidence and self-efficacy of the study participants in coping with the challenges and stresses of delivery of healthcare services. The scale consisted of 10 items and each item was placed on a 1–4 Likert scale, ranging from 1 (strongly disagree) to 4 (strongly agree). The total score for this section could range from 10 to 40, with a higher score indicating a greater confidence and self-efficacy. We stratified the final scores into three categories: (1) Low (scores between 10 and 20); (2) Moderate (scores between 21 and 30); and (3) High (scores between 31 and 40). The Cronbach’s alpha coefficient of internal consistency for reliability at pre- and post-testing was calculated at 0.87.

The third section of our research instrument was designed to assess insomnia. This section was architected based on the 1993 Insomnia Severity Index (ISI) instrument (Morin, 1993). This section comprised of seven items. The Likert scale for each item ranged from the value of 0 (none/not at all interfering) to the value of 4 (very severe or very much interfering). We stratified the final scores for this section into four categories: (1) No clinically significant insomnia (scores between 0 and 7); (2) Sub-threshold insomnia (scores between 8 and 14); (3) Clinical insomnia at moderate severity (scores between 15 and 21) and finally (4) Clinical insomnia at severe level (scores 22–28). The Cronbach’s alpha coefficient of internal consistency reliability for pre-testing was 0.89, while this value was 0.90 for post-testing. Finally, the fourth section of our research instrument was designed to assess depression, anxiety, and stress of the study participants. We adopted the Depression Anxiety Stress (DASS) instrument (Lovibond and Lovibond, 1995). This section contained 21 items; each item was given a range of 0–3 on the Likert scale, with the value 0 indicated “not all/never” while the value of 3 suggested “very must/most of the time.” The final scores were classified into five categories according to the recommended cut-off scores for conventional severity labels (Lovibond and Lovibond, 1995; Table 2). The Cronbach’s alpha coefficient of internal consistency for reliability at pre-testing was calculated at 0.96, while for post-testing, this value was 0.94. Questionnaires was translated into Khalkha dialect by one of the researchers with proficiency in English language and Khalkha dialect. The Khalkha translated version was evaluated by a mental healthcare professional for content validity and comprehensibility. Finally, two nursing professionals, independent of each other, one residing in the United States and the other in Mongolia, evaluated the final version of our research instrument for its content validity.

**TABLE 2:** Classification of depression anxiety stress scores according to the conventional cut-off points established by Lovibond and Lovibond (1995).

Levels of symptoms manifestation	Depression symptoms score	Anxiety symptoms score	Stress symptoms score
Normal	0–9	0–7	0–14
Mild	10–13	8–9	15–18
Moderate	14–20	10–14	19–25
Severe	21–27	15–19	26–33
Extremely severe	28≤	20≤	34≤

### Data Collection

This study was approved by the Ethical Board of the Mongolian National University of Medical Sciences (#2021/3-05). We approached the director of each participating hospital and obtained his/her approval before implementing our study. The director at each hospital assigned a research assistant to our study. We used the Google forms (A link to access our research instrument is available upon request) to distribute the research instrument to the 1,085 healthcare professionals who had consented to participate in our study. At each hospital, one of the members of the research team and the designated research assistant assumed the responsibility for the distribution of the research instrument and collection of data. Study participants completed the research instruments at a time convenient to them and returned their anonymously completed instruments to the research assistant. Data were collected from April 1st through June 30, 2021.

### Statistical Analysis

We used descriptive statistics to summarize the demographic characteristics of the study participants and the outcomes of ISI, GSES, and DASS assessments. Study participants were categorized by their professional title and differences in frequency distributions of the measured symptoms, perceived self-efficacy, depression, stress, and anxiety were assessed using the Kruskal–Wallis statistical test of significance. For variables that reached the level of statistical significance, we applied Dunn-Bonferroni *post hoc* method.

We then proceeded with applying the multinominal logistic regression statistical technique to determine the variables that best were associated with the risk of depression, anxiety, and stress during the peak response to the patient load. Due to the collinearity of the three responses (anxiety, stress, and depression), we developed the “Pandemic Response Symptoms (PaReSy).” Study participants, based on their reported responses, were categorized into “Normal Response Symptoms” group or “Mild/Moderate Response Symptoms” or “Severe/Extremely Severe” groups (Table 3). In developing the best fitted model, we also dichotomized the level of self-efficacy by collapsing the low and moderate levels. This approach was justified because of the low number of persons in the stratum of low self-efficacy in every category of healthcare profession; similarly, we collapsed the two strata of moderate and severe insomnia into one category because of the low number of persons in the severe insomnia across the groups of healthcare professionals. Finally, the variable age was categorized in to three groups: 20–29, 30–39, and 40 and older. We proceeded with estimating the individual effect of each variable, gender, age, education, professional classification, years of work experience, having children, self-efficacy, and insomnia on PaReSy/outcome. Variables with a *P*-value < 0.10 from the univariate analyses were considered as candidate variables. Interaction between the two variables, insomnia, and self-efficacy, on the outcome also was tested at *P* ≤ 0.10. The final model contained only variables that were significant at *P* ≤ 0.05. All statistical tested were two-sided and analysis were performed using SPSS Statistics 21.0 software.

**TABLE 3:** Classification of study participants by their reported levels of depression, anxiety, and stress.

Pandemic response symptoms	Depression	Anxiety	Stress	DASS^1^
Normal	0–9	0–7	0–14	0–30
Mild/moderate	10–20	8–14	15–25	31–59
Severe/extremely severe	21≤	15≤	26≤	60≤

*^1^DASS, Depression Anxiety Stress Scale.*

## Results

Of the total of 1,085 research instruments that were distributed, 965 (88.9%) were completed and returned (Figure 1). Of these 965 completed instruments, 881 (91.3%) were completed and returned by female healthcare professionals. Most of the respondents (79.6%, *n* = 671) were between ages of 20 and 39 years (Table 1). About 64% (*n* = 618) of them had completed their baccalaureate academic training in medical sciences. Stratification of the study participants by their professions, categorize 51% (*n* = 494) as nursing professionals, 21.8% (*n* = 210) as physicians; the remaining 27% (*n* = 261) were healthcare professionals with various technical expertise (Table 4).

**TABLE 4:** Demographic characteristics of the healthcare workers (*n* = 965).

Demographic characteristics	Frequency	Percentage (%)
**Gender**		
Female	881	91.3
Male	84	8.7
**Age in decades**		
20–29	326	33.8
30–39	345	35.8
40–49	200	20.7
50–59	92	9.5
60–69	2	0.2
**Education**		
High school	270	28.0
4-year baccalaureate academic training	618	64.0
More than 4-year academic training	77	8.0
**Professional classification**		
Nursing	494	51.2
Attending staff physician	210	21.8
Physician in-training	102	10.5
Laboratory technician	57	5.9
Sterilization technician	58	6.0
Radiology technician	22	2.3
Midwives	22	2.3
**Work experience (years)**		
1–5	381	39.4
6–10	207	21.5
11–15	126	13.1
More than 15	251	26.0
**Have children**		
Yes	770	79.8
No	195	20.2

The distribution of prevalence of anxiety, and perception of self-efficacy among different groups of healthcare providers reached the level of statistical significance (Table 5). Results from Dunn-Bonferroni *post hoc* statistical technique, suggested the level of anxiety (χ^2^ = 75.008, SE ± 22.895) was statistically significantly different between the nursing professionals and staff physicians, with the nursing professionals experiencing a higher level of anxiety (*P* = 0.001). While the level of anxiety among physicians in-training was statistically significantly higher than the nursing professionals (χ^2^ = 89.187, SE ± 30.225) (*P* = 0.003); finally, results of our statistical analysis suggested that the level of anxiety among radiologist technicians was higher compared with the physicians in-training (χ^2^ = 130.143 SE ± 65.328) (*P* = 0.046).

**TABLE 5:** Distribution of insomnia, self-efficacy, depression, anxiety, and stress by profession.

Outcome	Nurses N (%)	Staff physician N (%)	Physician in training N (%)	Laboratory technician N (%)	Sterilization technicians N (%)	Radiology technicians N (%)	Midwives N (%)	*P*-value
Self-efficacy								χ^2^ = 12.271, *P* = 0.015
Low	4 (0.8)	5 (2.4)	2 (2.0)	1 (1.8)	–	–	2 (3.4)	
Moderate	397 (80.4)	162 (77.1)	83 (81.4)	44 (77.2)	17 (77.3)	16 (72.7)	41 (70.7)	
High	93 (18.8)	43 (20.5)	17 (16.7)	12 (21.1)	5 (22.7)	6 (27.3)	15 (25.9)	
Insomnia								χ^2^ = 10.354, *P* = 0.053
No insomnia	163 (33.0)	80 (38.1)	34 (33.3)	27 (47.4)	9 (40.9)	7 (31.8)	16 (27.6)	
Subthreshold	216 (43.8)	90 (42.9)	45 (44.1)	25 (43.9)	11 (50.0)	9 (40.9)	32 (55.2)	
Moderate insomnia	92 (18.6)	33 (15.7)	20 (19.6)	5 (8.8)	2 (9.1)	6 (27.3)	9 (15.5)	
Severe insomnia	23 (4.6)	7 (3.3)	3 (2.9)	–	–	–	1 (1.7)	
Depression								χ^2^ = 5.071, *P* = 0.280
Normal	227 (46.0)	112 (53.3)	41 (40.2)	29 (50.9)	11 (50.0)	12 (54.5)	28 (48.3)	
Mild	87 (17.5)	40 (19.0)	20 (19.6)	8 (14.0)	2 (9.1)	3 (13.6)	11 (19.0)	
Moderate	119 (24.1)	41 (19.5)	26 (25.5)	13 (22.8)	8 (36.4)	5 (22.7)	17 (29.3)	
Severe	31 (6.3)	10 (4.8)	7 (6.9)	3 (5.3)	1 (4.5)	1 (4.5)	1 (1.7)	
Extremely severe	30 (6.1)	7 (3.3)	8 (7.8)	4 (7.0)	–	1 (4.5)	1 (1.7)	
Anxiety								χ^2^ = 16.064, *P* = 0.003
Normal	127 (25.7)	77 (36.7)	36 (35.3)	19 (33.3)	7 (31.8)	5 (22.7)	17 (29.3)	
Mild	48 (9.7)	17 (8.1)	8 (7.8)	3 (5.3)	1 (4.5)	1 (4.5)	6 (10.3)	
Moderate	133 (26.9)	53 (25.2)	34 (33.3)	17 (29.8)	9 (40.9)	6 (27.3)	10 (17.2)	
Severe	71 (14.4)	33 (15.7)	9 (8.8)	7 (12.3)	1 (4.5)	3 (13.6)	13 (22.4)	
Extremely severe	115 (23.3)	30 (14.3)	15 (14.7)	11 (19.3)	4 (18.2)	7 (31.8)	12 (20.7)	
Stress								χ^2^ = 4.446, *P* = 0.349
Normal	311 (63.0)	144 (68.6)	58 (56.9)	37 (64.9)	18 (81.8)	14 (63.6)	38 (65.5)	
Mild	68 (13.8)	32 (15.2)	21 (20.6)	7 (12.3)	–	3 (13.6)	12 (20.7)	
Moderate	53 (10.7)	18 (8.6)	11 (10.8)	6 (10.5)	2 (9.1)	3 (13.6)	5 (8.6)	
Severe	46 (9.3)	11 (5.2)	9 (8.8)	5 (8.8)	2 (9.1)	2 (9.1)	2 (3.4)	
Extremely severe	16 (3.2)	5 (2.4)	3 (2.9)	2 (3.5)	–	–	1 (1.7)	

Differences in perception of self-efficacy also reached the level of statistical significance. Results from Dunn-Bonferroni *post hoc* statistical analysis suggested that physicians in training had lower self-efficacy perception compared to the nursing professionals (χ^2^ = 97.815, SE ± 29.761) (*P* = 0.001), or midwives (χ^2^ = 101.104, SE ± 45.002) (*P* = 0.026), or staff physicians (χ^2^ = 93.720, SE ± 33.026) (*P* = 0.005); however, perception of self-efficacy of physicians in-training was statistically significantly higher than the sterilization technicians (χ^2^ = 146.454, SE ± 64.326) (*P* = 0.023).

Results from the multinominal logistic regression yielded healthcare professionals in the age category of 40 or older were less likely (OR = 0.677, 95% CI = 0.461–0.993, *P* = 0.046) to experience mild/moderate level of PaReSy relative to the younger healthcare providers (Table 6). Although, we did not detect a statistically significant association between age group and severe/extremely severe PaReSy (Table 6). Quite interestingly, we did not detect any statistically significant association between gender, or education or family structure (having children or not) and the categories of PaReSy (Table 6). Results from the multinominal logistic regression analysis, yielded that staff physicians relative to the nursing professionals were less likely to experience mild/moderate level of PaReSy (OR = 0.702, 95% CI = 0.487–1.012, *P* = 0.058) or severe/extremely severe level of PaReSy (OR = 0.494, 95% CI = 0.288–0.847, *P* = 0.010). Perception of self-efficacy was protective against experiencing mild/moderate PaReSy (OR = 0.948, 95% CI = 0.911–0.988, *P* = 0.011) or severe/extremely severe PaReSy (OR = 0.911, 95% CI = 0.861–0.963, *P* = 0.001).

**TABLE 6:** Results of multinominal logistic regression.

	Mild/Moderate PaReSy^1^ Referent: Normal PaReSy			Severe/Extremely PaReSy Referent: Normal PaReSy		
	OR^2^	95% CI^3^	*P*-value	OR	95% CI	*P*-value
**Age**						
30–40 vs. 20–29	1.157	0.812–1.647	0.419	0.986	0.576–1.688	0.959
41≤ vs. 20–29	0.677	0.461–0.993	0.046	1.069	0.618–1.850	0.810
**Gender**						
Male vs. female	1.338	0.808–2.215	0.258	1.072	0.498–2.309	0.859
**Education**						
Diploma vs. bachelor	1.140	0.808–1.609	0.455	1.401	0.860–2.283	0.176
Master≤ vs. bachelor	1.430	0.812–2.520	0.216	1.009	0.432–2.353	0.984
**Profession**						
Staff physicians vs. nurses	0.702	0.487–1.012	0.058	0.494	0.288–0.847	0.010
Laboratory tech vs. nurses	0.931	0.512–1.693	0.814	0.727	0.313–1.690	0.459
Physicians in training vs. nurses	0.919	0.513–1.645	0.776	0.515	0.204–1.303	0.161
Sterilization tech vs. nurses	1.128	0.702–1.813	0.618	0.753	0.383–1.481	0.411
Midwives vs. nurses	0.642	0.243–1.698	0.372	0.902	0.277–2.937	0.864
Radiology tech vs. nurses	1.090	0.432–2.752	0.855	0.777	0.204–2.960	0.711
**Children**						
Yes vs. No	1.247	0.876–1.774	0.221	1.171	0.722–1.900	0.521
**Perceived self-efficacy**						
Yes vs. No	0.948	911–0.988	0.011	0.911	0.861–0.963	0.001
**Insomnia**						
Sub-threshold vs. No	4.075	2.952–5.626	0.000	19.226	7.541–49.016	0.000
Moderate vs. No	6.826	4.201–11.093	0.000	93.273	34.747–250.379	0.000
Severe vs. No	5.560	1.663–18.590	0.005	265.649	65.836–1071.888	0.000

*^1^PaReSy, Pandemic Response Symptoms; ^2^OR, Odds ratio; ^3^95% CI, 95% confidence interval.*

Finally, we detected statistically significant association between insomnia and PaReSy (Table 6). Within each stratum of insomnia, the risk of experiencing PaReSy increased almost linearly both in the category of mild/moderate PaReSy and in the category of severe/extremely severe PaReSy, although the confidence interval was large in the category of PaReSy because of the relatively small numbers. Individuals who had reported sub-threshold levels of insomnia were almost four times more likely (OR = 4.07, 95% CI = 2.95–5.66, *P* = 0.000) to experience mild/moderate levels of PaReSy or severe/extremely severe PaReSy (OR = 19.23, 95% CI = 7.54–49.02, *P* = 0.000) relative to individuals who had reported on no insomnia. The risk of experiencing mild/moderate PaReSy increased to slightly more than 6-fold for individuals who had reported moderate insomnia (OR = 6.83, 95% CI = 4.20–11.09, *P* = 0.000), while for others moderate insomnia increased the risk of (OR = 93.27, 95% CI = 34.75–250.38, *P* = 0.000) PaReSy to the level of severe/extremely severe; this risk was more than 200 fold (OR = 265.65, 95% CI = 65.84–1071.89, *P* = 0.000) for individuals who had experienced severe insomnia.

## Discussion

Immediately after the WHO declaration of global COVID-19 pandemic on March 11, 2020, the government of Mongolia implemented strict travel and other public health measures across the country. In consequence, the public health impact of COVID-19 pandemic in Mongolia was less than other countries such as the United States; yet the healthcare infrastructure and the healthcare workforce in Mongolia experienced unprecedented strains in the delivery of healthcare services while facing with shortage of personal protective equipment and effective clinical therapeutic interventions. During the peak of the pandemic, many of our colleagues and frontline healthcare providers were experiencing various signs and symptoms of depression and psychological duress. Our professional ethics obligated us to systematically assess, document and report on the mental health and well-being of the Mongolian healthcare professionals. Although the concern about mental health of healthcare professionals was global, the decline in mental health of healthcare professionals in countries with limited number of healthcare providers and infrastructures became a paramount concern (Barzilay et al., 2020; Teixeira et al., 2020; Barua et al., 2021).

We constructed the instrument of Pandemic Response Symptoms (PaReSy) which captured stress, depression, and anxiety. Our data suggested that age was a protective factor against experiencing PaReSy; we attribute this finding to the work and life experiences of older healthcare professionals which enabled them to better cope with the stress of responding to the pandemic demands. Results are consistent with previous studies which found junior healthcare workers (HCW) suffer more from work-related stress (Dosil et al., 2020; Margaretha et al., 2020; Vizheh et al., 2020; Danet, 2021; Lee et al., 2021).

Our findings suggest that the nursing professionals were more likely to experience PaReSy relative to the staff physicians. We attribute the susceptibility of nurses to PaReSy to their role and responsibilities in providing continuous healthcare services and having more frequent interactions and deeper communications with patients; furthermore, the Mongolian healthcare work force, especially the nursing workforce, is relatively young; many of them had never experienced a sudden and precipitous increase in the number of patients requiring intensive and critical care. Results of our analysis strongly suggest that low perception of self-efficacy increased susceptibility to PaReSy. Many of the younger nursing staff had coping difficulties with the loss of their patients; we believe that many of the nursing staff internalized losses of their patients and therefore had reported their self-efficacies as low in dealing with the demands of the pandemic. Studies have been revealing high prevalence rates of depression, anxiety, insomnia and loss of self-efficacy among HCWs, treating infected patients (Vagni et al., 2020; Yıldırım and Özaslan, 2021). Conversely, better self-efficacy may play an important role in reducing anxiety and stress (Simonetti et al., 2021). Also, this study found that the prevalence of anxiety, sleep disorders and low levels of self-efficacy was consisted with Italian nurses providing care to patients with suspected or confirmed COVID-19.

Finally, our findings suggested that insomnia statistically significantly increased the susceptibility of the healthcare workforce to PaReSy. During the peak of the pandemic, many of the healthcare professionals, but particularly the nursing staff, were required to work beyond the international standard of 8-h per day. The size of Mongolian healthcare workforce is relatively small, the sudden increase in the demand for healthcare services offered no choice for the Mongolian Health Ministry but to mandate continuous and long workhours from the healthcare workforce, but particularly from the nursing professionals. The changes in workhours and separation from family members were compounded with the limited supplies of PPE, medications and/or the other needed medical technological interventions to treat patients. Additionally, healthcare providers in Mongolia, particularly those in the frontline, were concerned about their own wellbeing because of being at high risk of exposure to the COVID-19 virus. Findings from a meta-analysis of 46 studies, well documented that healthcare workers experienced anxiety and mental duress about their personal safety because of inadequate PPE, insufficient resources, and inconsistent information (Billings et al., 2021).

Our finding is consistent with previous studies which found that poor sleep quality was common during the pandemic and was associated with a 2–3 times the risk of reporting state anxiety, moderate depression, and stress. In addition, insomnia has been suggested to cause depression and/or anxiety disorders (Huang and Zhao, 2020; Zhang et al., 2020; Varma et al., 2021). Anxiety and sleep disorders have a great impact on the psychophysical health of nurses, affecting professional performance and patient safety (Simonetti et al., 2021). Moreover, our results are consistent with insomnia being a risk factor for the development of anxiety disorders (Barakat et al., 2016; Albasheer et al., 2020; Hsu and Chang, 2020; Barua et al., 2021).

Incidence of COVID-19, about 24 months after WHO declaration of COVID-19 pandemic has declined in Mongolia. This decline is attributed to the heroic work of the healthcare providers across the spectrum of professional expertise, the strict mandates enforced by the government of Mongolia, and the support of the Mongolian community by respecting and observing the public health restrictions. However, the uncertainties about the long-term impact of this pandemic on the healthcare workforce remain. For example, concerns have been raised about the older and therefore more experienced healthcare professional, particularly the nursing professionals, leaving the healthcare force. Mongolian government has taken decisive actions such as training more healthcare professionals to alleviate the long-term impact of the COVID-19 pandemic. Although, this is the right move, other measures must be addressed through legislations and implementation of public policies and to increase the size and experience of healthcare workforce in Mongolia.

### Strengths and Limitations

The main strengths of our study were the inclusion of a spectrum of frontline healthcare workers and its relatively large sample size. Our study had two limitations. The cross-sectional design of our study did not permit us to adequately discern the reason for the observed various mental health conditions among the frontline healthcare providers. Additionally, we relied on self-reported data; the respondents in our study were not evaluated by professional mental healthcare providers, i.e., psychologists; therefore, it is likely that a fraction of the respondents could have been in the state of denial about their mental health conditions. However, the overarching objective of our study was to systematically and scientifically assess and document prevalence of various mental and psychological side effects of the COVID-19 pandemic among Mongolian healthcare professionals, which is the first step in developing constructive strategies in correcting a health concern and/or condition. Our study further shed the light on the impact of work conditions on mental health of healthcare professionals, particularly among low- and middle-income countries. Future longitudinal research should be implemented to clarify the changes in stressors on mental health and assessment of effective long-term coping strategies.

## Implications

Poor mental health negatively impacts both the healthcare workforce and the organization. Policymakers, administrators, and healthcare professionals need to work in tandem in reaching consensus to promote healthcare professionals’ psychological wellbeing.

## Conclusion

Nursing professionals and less experienced healthcare providers were at a greater risk for various psychological duress during the peak of COVID-19 pandemic. Policies, and administrative measures should be encouraged and implemented to support strengthening of personal skills of healthcare professionals when facing with difficult and stress causing situation at workplace.

## Data Availability Statement

The original contributions presented in the study are included in the article/supplementary material, further inquiries can be directed to the corresponding author.

## Ethics Statement

The studies involving human participants were reviewed and approved by the Ethical Board of the Mongolian National University of Medical Sciences (#2021/3-05). The patients/participants provided their written informed consent to participate in this study.

## Author Contributions

EY, BT, and DC translated the questionnaires. OB, OD, and EG evaluated the content validity and comprehensibility. EY, OB, DC, EG, O-eA, OP, BDa, TS, and BDo performed the data collections. BT, OB, and AS performed the data analysis and interpretation. BT, DC, and OB wrote the first draft of the manuscript. AS performed the review and editing. All authors have involved and contributed equally to the development of concepts, design, and definition of intellectual content, and discussed and approved the final version of the manuscript.

## Conflict of Interest

The authors declare that the research was conducted in the absence of any commercial or financial relationships that could be construed as a potential conflict of interest.

## Publisher’s Note

All claims expressed in this article are solely those of the authors and do not necessarily represent those of their affiliated organizations, or those of the publisher, the editors and the reviewers. Any product that may be evaluated in this article, or claim that may be made by its manufacturer, is not guaranteed or endorsed by the publisher.

## Abbreviations

DASS: Depression Anxiety Stress Scale
GSES: General Self-Efficacy Scale
ISI: Insomnia Severity Scale
UK: United Kingdom
HCW: Healthcare workers
WHO: World Health Organization
PaReSy: Pandemic Response Symptoms
PPE: Personal Protective Equipment
UNICEF: United Nations International Children’s Emergency Fund
MoH: Ministry of Health
MRCS: Mongolian Red Cross Society
UNPF: United Nations Population Fund.

## References

[B1] Albasheer O. B. Al Bahhawi T. Ryani M. Arishi A. M. Hakami O. M. Maashi S. M. (2020). Prevalence of insomnia and relationship with depression, anxiety and stress among Jazan University students: A cross-sectional study. *Cogent Psychol.* 7:1789424.

[B2] Barakat D. Elwasify M. Elwasify M. Radwan D. (2016). Relation between insomnia and stress, anxiety, and depression among Egyptian medical students. *Middle East Curr. Psychiat.* 23 119–127. 10.1097/01.xme.0000484345.57567.a9

[B3] Barua L. Zaman M. S. Omi F. R. Faruque M. (2021). Psychological burden of the COVID-19 pandemic and its associated factors among frontline doctors of Bangladesh: a cross-sectional study. *F1000Research* 9:1304. 10.12688/f1000research.27189.3 33447383 PMC7783536

[B4] Barzilay R. Moore T. M. Greenberg D. M. DiDomenico G. E. Brown L. A. White L. K. (2020). Resilience, COVID-19-related stress, anxiety and depression during the pandemic in a large population enriched for healthcare providers. *Translat. Psychiat.* 10 1–8. 10.1038/s41398-020-00982-4 32820171 PMC7439246

[B5] Buchan J. O’May F. Dussault G. (2013). Nursing workforce policy and the economic crisis: a global overview. *J. Nurs. Scholars.* 45 298–307. 10.1111/jnu.12028 23656542

[B6] Burns N. Grove S. K. (2010). *Understanding nursing research*: Building an evidence-based practice. Berlin: Elsevier Health Sciences.

[B7] Billings J. Ching B. C. F. Gkofa V. Greene T. Bloomfield M. (2021). Experiences of frontline healthcare workers and their views about support during COVID-19 and previous pandemics: A systematic review and qualitative meta-synthesis. *BMC Health Serv. Res.* 21:1–17. 10.1186/s12913-021-06917-z 34488733 PMC8419805

[B8] Chimeddorj B. Byambatsogt G. Ryenchindorj E. (2021). SARS-CoV-2 seroprevalence in Mongolia: Results from a national population survey. *Lancet Regional Health Western Pacific* 17:100317. 10.1016/j.lanwpc.2021.100317 34841381 PMC8609908

[B9] Covid-19 Situation update, Mongolia Update#12 (2021). *International Federation of Red Cross.* Available online at: https://go.ifrc.org/reports/14436 (accessed June 22, 2021).

[B10] Covid-19 Situation Report for Mongolia #58 (2021). *Ministry of Health, Mongolia. As of 13 June 2021.* Available online at: https://www.who.int/mongolia/internal-publications-detail/covid-19-situation-report-for-mongolia-58 (accessed June 13, 2021)

[B11] Covid-19 Situation Report for Mongolia #62 (2021). *Ministry of Health, Mongolia. 12 July 2021.* Available online at: https://www.who.int/mongolia/internal-publications-detail/covid-19-situation-report-for-mongolia-62 (accessed July 12, 2021)

[B12] Danet A. D. (2021). Psychological impact of COVID-19 pandemic in Western frontline healthcare professionals. A systematic review. *Med. Clín.* 7 449–458. 10.1016/j.medcle.2020.11.003 PMC7972644 33758782

[B13] Dosil M. Ozamiz-Etxebarria N. Redondo I. Picaza M. Jaureguizar J. (2020). Psychological symptoms in health professionals in Spain after the first wave of the COVID-19 pandemic. *Front. Psychol.* 11:60612. 10.3389/fpsyg.2020.606121 33391125 PMC7775406

[B14] Gené-Badia J. Gallo P. Hernández-Quevedo C. García-Armesto S. (2012). Spanish health care cuts: penny wise and pound foolish? *Health Policy* 106 23–28. 10.1016/j.healthpol.2012.02.001 22494526

[B15] Huang Y. Zhao N. (2020). Generalized anxiety disorder, depressive symptoms and sleep quality during COVID-19 outbreak in China: a web-based cross-sectional survey. *Psychiat. Res.* 288:112954. 10.1016/j.psychres.2020.112954 PMC7152913 32325383

[B16] Hsieh F. Y. (1989). Sample size tables for logistic regression. *Statist. Med.* 8 795–802. 10.1002/sim.4780080704 2772439

[B17] Hsu Y. W. Chang C. P. (2020). Stress of life events and anxiety as mediators of the association between insomnia and triglycerides in college students. *J. Am. College Health* 2020 1–7. 10.1080/07448481.2020.1799805 32790499

[B18] Hopman J. Allegranzi B. Mehtar S. (2020). Managing COVID-19 in low-and middle-income countries. *JAMA* 323 1549–1550. 10.1001/jama.2020.4169 32176764

[B19] Lee J. Lee H. J. Hong Y. Shin Y. W. Chung S. Park J. (2021). Risk perception, unhealthy behavior, and anxiety due to viral epidemic among healthcare workers: the relationships with depressive and insomnia symptoms during COVID-19. *Front. Psychiat.* 12:358. 10.3389/fpsyt.2021.615387 33815164 PMC8017167

[B20] Lipstein S. H. Kellermann A. L. (2016). Workforce for 21st-century health and health care. *JAMA* 316 1665–1666. 10.1001/jama.2016.13715 27669060

[B21] Lovibond P. F. Lovibond S. H. (1995). The structure of negative emotional states: Comparison of the Depression Anxiety Stress Scales (DASS) with the Beck Depression and Anxiety Inventories. *Behav. Res. Therapy* 33 335–343. 10.1016/0005-7967(94)00075-u 7726811

[B22] Margaretha S. E. P. M. Effendy C. Kusnanto H. Hasinuddin M. Madura S. N. H. (2020). Determinants psychological distress of Indonesian health care providers during covid-19 pandemic. *Profession* 116 1–17.

[B23] Mattei A. Fiasca F. Mazzei M. Abbossida V. Bianchini V. (2017). Burnout among healthcare workers at L’Aquila: its prevalence and associated factors. *Psychol. Health Med.* 22 1262–1270. 10.1080/13548506.2017.1327667 28503931

[B24] Morin C. M. (1993). *Insomnia: Psychological assessment and management.* New York, NY: Guilford press.

[B25] Mongolia Center for Health Development (2019). *Health Indicators 2019.* Available online at: http://hdc.gov.mn/media/uploads/2020-08/2019-eruul_mendin_uzuulelt_MU_mail.indd_2020_______7___21final.pdf (accessed May 21, 2020)

[B26] Mongolia Center for Health Development (2021). *Health Statistical Information.* Available online at: http://hdc.gov.mn/media/uploads/2021-10/2021-09_Sariin_medee.pdf (accessed May 21, 2021)

[B27] Reeves A. McKee M. Basu S. Stuckler D. (2014). The political economy of austerity and healthcare: Cross-national analysis of expenditure changes in 27 European nations 1995–2011. *Health Policy* 115 1–8. 10.1016/j.healthpol.2013.11.008 24315493

[B28] Simonetti V. Durante A. Ambrosca R. Arcadi P. Graziano G. Pucciarelli G. (2021). Anxiety, sleep disorders and self-efficacy among nurses during COVID-19 pandemic: A large cross-sectional study. *J. Clin. Nurs.* 30 1360–1371. 10.1111/jocn.15685 33534934 PMC8012992

[B29] Situation Report for Covid-19 (2020). *Ministry of Health, Mongolia.* Available online at: https://mrc-ide.github.io/global-lmic-reports/MNG (accessed June 23, 2020).

[B30] Situation Report #4 (2020). *2020 on COVID-19 response, UNFPA MONGOLIA.* Available online at: https://www.unfpa.org/resources/covid-19-situation-report-no-4-unfpa-mongolia (accessed October 09, 2020)

[B31] Schwarzer R. Jerusalem M. (1995). Generalized self-efficacy scale. Measures in health psychology: A user’s portfolio. *Causal Control Beliefs* 1 35–37. 10.1097/PHM.0b013e318230fb68 21975679

[B32] Teixeira C. F. D. S. Soares C. M. Souza E. A. Lisboa E. S. Pinto I. C. D. M. Andrade L. R. D. (2020). A saúde dos profissionais de saúde no enfrentamento da pandemia de Covid-19. *Ciência Saúde Coletiva* 25 3465–3474. 32876270 10.1590/1413-81232020259.19562020

[B33] Vagni M. Maiorano T. Giostra V. Pajardi D. (2020). Coping with COVID-19: emergency stress, secondary trauma and self-efficacy in healthcare and emergency workers in Italy. *Front. Psychol.* 11:566912. 10.3389/fpsyg.2020.566912 33013603 PMC7494735

[B34] Varma P. Burge M. Meaklim H. Junge M. Jackson M. L. (2021). Poor Sleep Quality and Its Relationship with Individual Characteristics, Personal Experiences and Mental Health during the COVID-19 Pandemic. *Int. J. Environ. Res. Public Health* 18:6030. 10.3390/ijerph18116030 34205195 PMC8200012

[B35] Vizheh M. Qorbani M. Arzaghi S. M. Muhidin S. Javanmard Z. Esmaeili M. (2020). The mental health of healthcare workers in the COVID-19 pandemic: A systematic review. *J. Diabet. Metab. Disord.* 19 1967–1978. 10.1007/s40200-020-00643-9 PMC7586202 33134211

[B36] World Health Organization (2021a). *Health Workforce.* Available online at: https://www.who.int/health-topics/health-workforce#tab=tab_1 (accessed April 21, 2021)

[B37] World Health Organization (2021b). *COVID-19 Strategic Preparedness and Response Plan. 2021 (SPRP 2021).* Available online at: https://www.who.int/publications/i/item/WHO-WHE-2021.02 (accessed February 24, 2021)

[B38] Yıldırım M. Özaslan A. (2021). Worry, severity, controllability, and preventive behaviours of COVID-19 and their associations with mental health of Turkish healthcare workers working at a pandemic hospital. *Int. J. Mental Health Addic.* 2021 1–15. 10.1007/s11469-021-00515-0 33686345 PMC7928196

[B39] Zhang W. R. Wang K. Yin L. Zhao W. F. Xue Q. Peng M. (2020). Mental health and psychosocial problems of medical health workers during the COVID-19 epidemic in China. *Psychother. Psychosomat.* 89 242–250. 10.1159/000507639 32272480 PMC7206349

